# Twenty-Fourth Annual Meeting of the American Medical Association

**Published:** 1873-06

**Authors:** 


### TWENTY-FOURTH ANNUAL MEETING OF THE AMERICAN MEDICAL ASSOCIATION.

[We are indebted for the following abstract to the *Detroit Review of Medicine*:]

### FIRST DAY.

The twenty-fourth annual meeting of the Association was opened Thursday morning, May 8th, at eleven o'clock, in the Masonic Hall, at St. Louis, Mo. About five hundred physicians met at the appointed hour, when Dr. Yandell, Louisville, the retiring President, called the meeting to order. After prayer, Dr. John S. Moore, an old, prominent physician of St. Louis, welcomed the visitors in a short speech, which was well received.

Dr. Thomas M. Logan, of Sacramento, Cal., the President for 1873, was then introduced by the retiring President, and received with applause.

ENTERTAINMENTS.

The local committee reported the following arrangements for the pleasure of visiting members:

This evening there will be a *soirée musicale* at Mercantile Library Hall, commencing at eight o'clock. A band has been engaged, and arrangements made with Pezolt for refreshments. The wives and daughters of the physicians are expected to be present.

To-morrow evening there will be held a *levee* at the residence of Colonel J. L. D. Morrison, corner of Locust street and Leffingwell avenue.

On Thursday evening Dr. J. J. Woodward, U. S. A., of Washington, will, by special request of the Committee of Arrangements, deliver the Toner lecture at Masonic Hall. The announcement was received with great applause.

On Friday afternoon the visitors will view LaFayette Park, Tower Grove, and Shaw's Garden. Carriages to leave the hall at two o'ctock.

SPECIAL ORDERS.

A letter from Dr. Woodward, U. S. A., on the organization of the medical corps of the army, was made the special order for to-morrow.

An invitation was received from the St. Louis Mutual Life Insurance Company to visit their magnificent building.

Chauncy I. Filley, postmaster of St. Louis, has placed an elegant mail box in the hall, and notified members that every facility would be afforded visiting delegates to receive and dispatch their letters without delay. (Applause.)

A special committee on ethics was appointed to report whether certain parties are worthy of full fellowship with the Association. Drs. N. S. Davis, E. L. Howard, H. F. Askew, D. W. Yandell, and J. M. Toner were appointed, and the list of disputed names referred to them.

President Logan then delivered the annual address, which was listened to with attention, although lengthy. He called attention to the impulse given to the California medical men by the meeting of the Association in San Francisco. Giving a short history of the Association, showing that American physicians and surgeons rank as high as any in Europe, and repudiating the idea of some Germans who signalized therapeutics as something hardly better than nihilism, and the practice of physic not much more than a "meditation on death," he continued as follows:

"Imbued with the conviction that the beginning of wisdom is the knowledge of ignorance, and conscious of the difficulties which on every hand beset him, the scientific physician explores cautiously, doubts judiciously, and determines slowly. But when he rejects the hastily conceived and immature speculations of the self-satisfied empiric, he does not stand idly by and let disease run its course unmodified. Knowing that the Creator has established certain relations between cause and effect, and that all the phenomena which we witness around us are the result of antecedents, and not of chance, he seeks to fathom the causes of diseases, and by his knowledge of their course, and of the dangers which threaten the life of the patient, at each stage of their progress he interferes to prevent, to control, and to counteract any untoward consequences, and, by the judicious employment of all the rational means at his command, among which pure air, food, and stimulants are included, he saves the patient from death. Now, deny that this treatment can be regarded as *nil* or *expectant;* it is positive, nay, active.

"Believing, as I do, that medicine is destined, if her votaries only prove true to their allegiance, to reach that 'Ultima Thule' in its history when the stigma of *uncertain* shall be wiped away from its deductions, and it shall take its rank among the *exact* sciences, I can not but think that so long as it may well be doubted if any fact or principle yet obtained in regard to therapeutic agency in resisting morbific influences, can claim the rigidity and universality of a positive law, so long must scientific medicine continue her unwearied efforts after truth through the realms of physical research—so long widen perpetually her range, through the vast compass of subjects with which it is linked, by the progress of science and the fluctuations of human requirements. * * * * * * *

"Having said this much in the hope of vindicating our profession, in its relations with modern science, from the charge of skepticism or inefficiency, I must, at the hazard of being tedious to you, and lest my motives be impugned, disclaim the slightest intention of taking any undue advantage, or committing the least injustice or a misrepresentation of what I am willing to believe are the honest convictions of those who differ from me, and who, doubtless, are possessed of a common interest in the honor and usefulness of our calling. Possibly I may have misconceived the import of the arguments I have just been combating, or I may have misapprehended their significance. But when I am made painfully observant of the irregular and imperfect system of training under which the medical student is educated in our country; when I am witness of the superficial qualifications attaching to the conferring of the diploma in some of our colleges, and especially when I hear that system and that practice not only defended, but advocated, I feel that I should prove recreant to the duties of the high position I now hold were I not to give a just expression of my disapprobation, lest silence might be construed into an endorsement of what I conceive to be a most dangerous and demoralizing doctrine. And I am the more strengthened and confirmed in this opinion when I reflect that this Association, whose glorious record I have just sketched, and whose name has already extended far beyond the continent of its origin, was organized chiefly to elevate and ennoble the medical profession.

"It is far from my purpose, however, to speak disparagingly of those leading minds in our ranks who have been and still are honorably engaged in teaching—professors who, tried by any standard which the older civilization of Europe may set up, are entitled to the fame they have honestly won, and to our lasting gratitude. These men have ever been among the foremost and most earnest in demanding that upon every son of America the blessing of education should be bestowed, and that the blessing be made as thorough as possible. Were it not that it might appear invidious, I could cite a long list of living, honorable physicians, from every State of our extended territory, fit successors of Chapman, and Stevens, and Drake, and Warren, and Moultrie, and Wellford, and Dickson, and Pitcher, and Pope, and other departed worthies; men whose reputation is bounded by no geographical limits, citizens of the universal republic of letters, who have labored in season and out of season, and on all occasions, in advocacy of the broadest and completest education. In the hands of such men—who may be styled the trustees of the Association, in this particular—I repose every confidence. We may safely acknowledge them to be fully competent to inaugurate the reformatory measures so imperiously required. Their experience as professors renders them peculiarly sensitive to the evils now existing, and to the urgent need for their removal. Their competency in every particular for the undertaking is undeniable, whether in the abstruse and comprehensive, or in the refined and æsthetic; whether in the profound and logical, or in the powerful and commanding; whether in practical wisdom, moral, international or civil, social or medical, in those arts which employ while they improve and bless the people; whether, in a word, in all that makes man useful, virtuous, and happy, and that prepares him for the service of his Creator on earth, or of his fellow-men, or of posterity."

The speaker then referred in glowing terms to the power Harvard University had had in elevating the character and value of the diploma that qualified them to practice. He referred them to the power that cultivated men would possess in bringing about such legislation as would tend to make the Association an instrument of public good, and glanced at the necessity of establishing State Boards of Health, with a Central Sanitary Bureau to be established by the general Government. Having again urged the necessity of some alteration in the workings of the organization, which he supported by weighty arguments, he concluded by saying:

"But, gentlemen, whatever course you may think proper to pursue, I am sure that your objects will be the advancement of science, the good of humanity, and the honor and glory of our beloved profession, which for a continuous period of more than two thousand years has numbered among its votaries many of the wisest and most beneficent of the long roll of sages and philanthropists. I feel, therefore, that I can not better conclude than by bidding you hearken to the utterances of Missouri's honored son, (Dr. C. A. Pope,) *clarum et venerabile nomen*, pronounced eighteen years ago before this Association, and now echoed back from his sepulchral couch, in the capital of France

"'On the eve of the battle of the Pyramids, Napoleon exclaimed: 'Soldiers! from the height of yon monuments forty centuries look down upon you.' Gentlemen, from the heights of past ages countless worthies of our God-like profession point and beckon to a goal more elevated than ever attracted legislators and conquerors, Solons and Cæsars!'"

At the conclusion of the above speech a vote of thanks was given Dr. Logan, and the speech referred to the Committee on Publications.

The delegates from different States were instructed to appoint one delegate from each State to act as a Nominating Committee.

### SECOND DAY.

The Association was called to order at a quarter to ten o'clock.

### NOMINATING COMMITTEE.

The President called for the members of the Nominating Committee. The Secretary called the roll of States, and received the name of one delegate from each State.

The Committee of Arrangements reported a number of names for permanent membership, and for seats in the convention by invitation. Owing to some informality, the report was referred back to the committee.

### DETROIT WANTS THE NEXT SESSION.

A communication was received from Detroit asking the Association to hold the next annual session in that city. Referred to the Nominating Committee.

Dr. Woodward, U. S. A., read a letter requesting the Association to petition Congress to aid the army medical corps in obtaining equal pay and promotion with the officers of the navy, etc.

Dr. Keller, of Louisville, presented the following resolutions, which were unanimously adopted:

*Resolved,* That, in the opinion of this Association, the rank of the medical officers of the army ought to be fully equal to that of the officers of any other staff corps, or of the medical corps of the navy. That we learn with regret that this is not the case, and that we regard with grave disapprovnl the odious discrimination thus made against a meritorious body of officers.

*Resolved*, That we look upon the law which prohibits promotion and appointments in the medical corps of the army as unwise and unjust, and that, in our opinion, it ought forthwith to be repealed.

*Resolved*, That a committee of five be appointed by the President, to memorialize Congress on this subject, and that each member of this Association pledges himself to use all his influence with the member of Congress from his own district in behalf of the object of these resolutions.

The President appointed as the committee, Dr. Keller, of Kentucky; Dr. Askew, of Delaware; Dr. N. S. Davis, Chicago, Ill.; Dr. John Murphy, Cincinnati, Ohio; Dr. J. M. Toner, Washington, D. C.

MEDICAL EDUCATION.

An abstract of the report of the Committee on Medical Education was presented by the chairman of the committee, Dr. Carson, of Ohio. The following extracts are from the report:

"It is within the knowledge of the whole profession that there is much dissatisfaction regarding the important question of medical education, and particularly with reference to the results of the agitation of it in this Association. There is reason to think that at the basis of this dissatisfaction there lies a misconception of the influences affecting medical education, and of the functions of this body pertaining thereto. A proper consideration of these influences will show that some of them impose limitations upon medical education which it is beyond the power of the profession or of this body to control in any great degree, and that from others are evolved the spirit and method of new teaching, which are much within the personal efforts of all physicians to direct and control, and, therefore, that the administrative agencies, under which we include every form of teaching, whether private or public, in office or college, are not responsible for failures of performance in the first direction, while in the latter they and all of us are largely so. These influences are classified as, first, those originating in the external relations of medicine, such as government, general education and culture; physical and economical necessities. Secondly, those originating in the internal relations of medicines, such as are involved in the study and practice of the accessory and direct sciences and arts of medicine. Third, there are certain conditions derivative from these in the operation of the administrative agencies which exert important effects upon the results of medical education." The report here treats upon the various topics as classified above, and concludes as follows: "We conceive, then, that by recognizing the fact that we have two classes of influences reacting on medical education; which we have called the external and internal, of all of which it may be said that they are the products of growth under certain definite and inherent impulses, with power of adaptation, modification, etc.; of the first of which it may be said that they are very stable elements; of the second, it may be said that they are moving with the swift velocity of modern science. We conceive, then, that the power and function of our medical administrative agencies, and of this Association, may be more clearly defined. It would diminish the responsibility placed upon schools in all matters pertaining to the external relation of medicine, while it would hold them to increased responsibility in all matters belonging to the subjective work of their position. It would show that this Association can only exert great influence by increasing its efficiency as a tribunal, before which these subjects may be brought for the exercise of that most potent influence, discussion; and not for that impotent purpose of expecting to increase legislative functions toward the teaching bodies. If amendatory action be desired for this and other good purposes, then we herewith advocate it." The report was referred to the Committee on Publication.

Dr. Moore, the chairman of the Committee on Prize Essays, reported only one production which was considered not worthy.

As delegates to the British Medical Association, were appointed: Drs. F. G. Smith, C. Wister, J. S. Cohen, Philadelphia; E. Warren, of Baltimore; L. C. Ives, of New Haven; E. Montgomery, of St. Louis; Drs. E. Sequin, F. Baker, and J. C. Hutchinson, of New York; and Dr. J. F. Noyes, of Detroit.

The Committee on Nominations then reported as follows:

*President*—Dr. J. M. Toner, District of Columbia.

*First Vice-President*—W. Y. Gadberry, of Mississippi.

*Second Vice President*—J. M. Keller, of Kentucky.

*Third Vice-President*—W. C. Husted, of New York.

*Fourth Vice-President*—L. D. Warner, of Massachusetts.

*Treasurer*—Dr. Casper Wistar, of Philadelphia.

*Librarian*—Wm. Lee.

*Committee on Libraries*—Johnson Elliott.

*Secretary*—Theodore McGraw.

*Committtee on Arrangements*—Dr. Brodie, chairman; James A. Brown, Morse Stewart, J. F. Noyes, E. W. Jenks, Henry F. Lyster, D. O. Farrand, Eugene Smith; all of Detroit.

*Committee on Prize Essays*—Drs. J. K. Johnson, A. Sager, H. Hitchcock, of Michigan; E. Andrews, Illinois; E. S. Gaillard, Kentucky.

*Committee on Publications*—Drs. F. G. Smith, W. B. Atkinson, D. Morrey Chester, of Pennsylvania; Wm. Lee, District of Columbia; Casper Wistar, Pennsylvania; H. F. Askew, Delaware; Alfred Stille, Pennsylvania.

Detroit was named for the next annual meeting of the Association.

The Treasurer's report showed a balance of $450.

Dr. S. N. Davis then proposed the following amendments to the Constitution:

"The delegates shall receive their appointments from permanently organized State medical societies, and such county and district medical societies as are recognized by representation in their respective State societies, and from the medical department of the army and navy of the United States.

"Each State, county, and district medical society entitled to representation, shall have the privilege of sending to the Association one delegate for every ten of its regular resident members, and one for every additional fraction of more than half that number. The medical staffs of the army and navy shall be entitled to four delegates each."

Dr. Brodie, of Detroit, proposed that a committee of one from each State be appointed, whose duty it shall be to revise the code of ethics, and report at the next annual meeting, but, at the suggestion of Dr. Woodward, the subject was referred to the Judicial Committee.

A communication was received inviting the members of the Association to visit the Institution for the Blind, which was accepted.

A report was received from Surgean Guoin, of the United States navy, on Medical Education, with reference to the United States hospitals, which was referred, without being read, to the section on practical medicine.

Drs. Gross and Jackson, the delegates from the last convention to the British Medical Association, also sent in a voluminous report, which was referred to the Committee on Publications.

Dr. Fred. Horner moved that a committee of one member from each of the original thirteen States of the Union be appointed, to report to the Centennial Celebration on the medical, surgical, and biographical literature of the period of 1776, as a tribute to the memory of Joseph Warren, Benj. Rush, Arthur Lee, General Hugh Mercer, and the other noble and patriotic physicians who aided to secure the American independence, which was carried unanimously.

Dr. Peck, of Iowa, offered the following resolutions:

WHEREAS, The reports of the surgeon-general of the United States army, as exhibited in volumes one and two of the first part of the Medical and Surgical History of the War of the Rebellion, have received a too limited circulation by reason of an insufficient issue of the same by Congress; therefore—

*Resolved*, That the President and Secretary of this Association be directed to petition Congress at the next session, in behalf of the profession, asking that the edition recently issued be reproduced in sufficient number to permit of general distribution to the members of the profession throughout the country.

*Resolved*, That the thanks of this Association are due and are hereby tendered to Congress for aiding thus far in developing and presenting to the profession the reports of the surgeon-general, as herein specified.

*Resolved*, That the thanks of this Association are hereby tendered the officers of the United States army, who have by sacrifice and labor been instrumental in placing before the profession the valuable information contained in volumes one and two, of the first part of the Medical and Surgical History of the War of the Rebellion.

The resolutions were adopted unanimously, after which the convention adjourned.

### THIRD DAY'S PROCEEDINGS.

The report of the Committee on the Nomenclature of Diseases elicited a prolonged discussion, which was ended by adopting the following resolutions offered by Dr. Woodward:

*Resolved,* That, in the opinion of this Association, it is inexpedient to adopt the nomenclature and classification presented by the majority of the Committee on Nomenclature at the meeting in Philadelphia.

*Resolved,* That a committee of three be appointed by the President, whose duty it shall be to communicate the foregoing resolution to the proper committee of the Royal College of Physicians of London, and to negotiate for the representation of the American Medical Association in the first decennial revision of their nomenclature.

Dr. Toner then offered the following on the same subject, which was also adopted:

*Resolved,* That, in the opinion of this Association, it would be an opportune occasion at the American Centennial of 1876, for holding an international medical congress, to consider, and, if practicable, adopt a uniform classification and nomenclature of diseases, to be used by the profession throughout the world.

The Committee on Ethics reported against allowing a representation of the Pathological Society, of Berks county, Penn., in this Association.

A lengthy discussion on the Secretary's salary was ended by the adoption of Dr. Keller's motion:

*Resolved,* That the sum of $300 be appropriated as an honorarium for the services of the Permanent Secretary, during the present year, provided such an amount remains in the treasury after paying necessary expenses.

The question of salary being disposed of, Dr. Bell, of Brooklyn, N.Y., submitted the following concerning the establishment of a National Sanitary Bureau.

*Resolved,* That in the judment of this Association the establishment of a national sanitary bureau, with relations to the general government similar to those of the bureaus of agriculture and education, is highly desirable as a means of promoting sanitary science and the protection of the public health.

*Resolved,* That this Association request of the United States Educational Bureau, to so extend the scope of its inquiry as to include vital diseases and mortuary statistics, in relation to local, meteorological and geological influences, and to disseminate the information so collected throughout the country.

This was referred to a Committee on Public Hygiene and State Medicine.

The report of the Committee on Medical Education was tabled after considerable stormy discussion.

REPORT OF SPECIAL COMMITTEE.

The committee appointed to advise and recommend some plan for securing a more complete report of the doings in the several sections of the Association, respectfully report the following resolutions, and recommend their adoption :

1. *Resolved,* That the Committee of Arrangements for each annual meeting of the Association are instructed to secure the services of a sufficient number of stenographic reporters to have one in attendance on the regular sessions of each of the sections in the afternoon, as well as during the general morning sessions; that the reports thus obtained be printed the same evening on slips, or proof-sheets, in sufficient number to supply all the members of the Association in attendance, early the following morning.

2. *Resolved,* That the necessary expenses incurred in carrying into effect the foregoing resolution shall be paid from the treasury of the Association, in the same manner as other bills relating to the publication of the transactions.

3. *Resolved,* That the acting Secretary of each section shall keep a file of so much of the reports as relate to the business of the section to which he belongs, and it shall be his duty to revise the same, carefully eliminating therefrom all that is unimportant, and arranging that which is found valuable and pertinent in proper form ; he shall transmtt the same to the Permanent Secretary, for the Committee of Publication, within twenty days after the adjournment of each session of the Association·

The report was adopted.

The following gentlemen were appointed Chairmen and Secretaries of sections for 1874:

1. Practice of Medicine, Materia Medica and Physiology—Dr. N. S. Davis, of Chicago, Chairman; and Dr. Frothingham, of Ann Arbor, Mich., Secretary.

2. Obstetrics and Diseases of Women and Children—Dr. Theo. Parvin, of Indianapolis, Chairman; and Dr. Montrose A. Pallen, of St. Louis, Secretary.

3. Surgery and Anatomy—Dr. S. D. Gross, of Philadelphia, Chairman; and Dr. Alonzo Garcilon, of Maine, Secretary.

4. Medical Jurisprudence, Chemistry, and Psychology—Dr. A. N. Talley, of South Carolina, Chairman; and Dr. E. L. Howard, of Maryland, Secretary.

State and Public Hygiene—Dr. A. N. Bell, of Brooklyn, Chairman; and Dr. A. B. Stuart of Winona, Minnesota, Secretary: F. A. Ross, Alabama; D. A. Linthicum, Arkansas; T. M. Logan, California; R. G. Buckingham, Colorado; B. H. Catlin, Connecticut; L. P. Bush, Delaware; F. Howard, District of Columbia; W. F. Westmoreland, Georgia; H. A. Johnson, Illinois; W. H. Myers, Indiana; J. J. Angear, Iowa; D. W. Stormont, Kansas; L. Rodgers, Kentucky; S. Fitch, Maine; James Stewart, Maine; H. J. Bowditch, Massachusetts; R. C. Kedzie, Michigan; A. B. Stuart, Minnesota; J. W. M. Shattuck, Mississippi; John M. Hogden, Missouri; J. H. Parsons, New Hampshire; E. M. Hunt, New Jersey; A. N. Bell, New York; U. A. B. Norcom, North Carolina; Wm. Clendenin, Ohio; A. M. Pollock, Pennsylvania; G. M. Lenore, Rock Island; R. A. Kinlech, South Carolina; W. K. Briggs, Tennessee; D. R. Wallace, Texas; J. H. Cabell, Virgfnia; James Broomfield, West Virginia; H. B. Strong, Wisconsin; A. F. Woodward, Vermont; S. M. Bemiss, Louisiana.

*Resolved*, That the Secretary of the Association be authorized to fill up all vacancies in the committee from the States and Territories.

Also, That Dr. A. N. Bell be President, and Dr. A. B. Stuart, Secretary.

COMMITTEE ON NECROLOGY.

Chairman, Dr. A. Sager, Ann Arbor, Michigan; and committee remain as last year, excepting Dr. Alonzo Garcilon, of

Maine, in place of Dr. D. McReese, deceased; Dr. W. McCabe, Kansas; J. W. H. Baker, Iowa; J. H. Wheeler, New Hampshire; ——— Milligan, Minnesota; W. M. Chambers, Illinois; G. Sutton, Indiana: A. J. Semmes, Georgia; B. A. Vaugh, Mississippi; George Mitchell, Ohio; J. J. Woodward, U. S. A.; N. Dodson, Wisconsin; L. P. Yandell, Sen., Kentucky.

JUDICIAL COMMITTEE.

Three years—Dr. Brodie, Mich.; N. S. Davis, Ills.; E. L. Howard, Maryland; Wm. O. Baldwin, Alabama; Dr. Dean, N.Y., J. P. Logan, Ga.

Two years—L. S. Joynes, Va.; R. N. Todd, Ind.; Askew, Del.; J. E. Morgan, D.C.; Daniel Lillie, N.J.; S. M. Benham, Penn.; Dr. Dunlap, Conn.

One year—Dr. Bartlett, Wis.; Powell, Ill.; Gale, Ky.; Moses, Mo.; Hughes, Iowa; Bemiss, La.; Cheever, Mass.

Dr. R. H. Storer, of Boston, was appointed chairman of committee to report on American, as compared with foreign winter cures.

